# Anticancer Effects of Fucoxanthin through Cell Cycle Arrest, Apoptosis Induction, and Angiogenesis Inhibition in Triple-Negative Breast Cancer Cells

**DOI:** 10.3390/molecules28186536

**Published:** 2023-09-09

**Authors:** Shade’ A. Ahmed, Patricia Mendonca, Samia S. Messeha, Karam F. A. Soliman

**Affiliations:** 1 Division of Pharmaceutical Sciences, Institute of Public Health, College of Pharmacy and Pharmaceutical Sciences, Florida A&M University, Tallahassee, FL 32307, USA; shade1.ahmed@famu.edu; 2 Department of Biology, College of Science and Technology, Florida A&M University, Tallahassee, FL 32307, USA; samia.messeha@famu.edu

**Keywords:** triple-negative breast cancer, fucoxanthin, cell cycle arrest, angiogenesis, apoptosis

## Abstract

The absence of progesterone receptors, estrogen receptors, and human epidermal growth factor receptor-2 restricts the therapy choices for treating triple-negative breast cancer (TNBC). Moreover, conventional medication is not highly effective in treating TNBC, and developing effective therapeutic agents from natural bioactive compounds is a viable option. In this study, the anticancer effects of the natural compound fucoxanthin were investigated in two genetically different models of TNBC cells: MDA-MB-231 and MDA-MB-468 cells. Fucoxanthin had a significant anticancer effect in both cell lines at a concentration range of 1.56–300 µM. The compound decreased cell viability in both cell lines with higher potency in MDA-MB-468 cells. Meanwhile, proliferation assays showed similar antiproliferative effects in both cell lines after 48 h and 72 h treatment periods. Flow cytometry and Annexin V-FITC apoptosis assay revealed the ability of fucoxanthin to induce apoptosis in MDA-MB-231 only. Cell cycle arrest analysis showed that the compound also induced cell cycle arrest at the G1 phase in both cell lines, accompanied by more cell cycle arrest in MDA-MB-231 cells at S-phase and a higher cell cycle arrest in the MDA-MB-468 cells at G2-phase. Wound healing and migration assay showed that in both cell lines, fucoxanthin prevented migration, but was more effective in MDA-MB-231 cells in a shorter time. In both angiogenic cytokine array and RT-PCR studies, fucoxanthin (6.25 µM) downregulated VEGF-A and -C expression in TNF-α-stimulated (50 ng/mL) MDA-MB-231, but not in MDA-MB-468 cells on the transcription and protein levels. In conclusion, this study shows that fucoxanthin was more effective in MDA-MB-231 TNBC cells, where it can target VEGF-A and VEGF-C, inhibit cell proliferation and cell migration, and induce cell cycle arrest and apoptosis—the most crucial cellular processes involved in breast cancer development and progression.

## 1. Introduction

Breast cancer is the most common cancer in women worldwide and the second most diagnosed cancer among women in the United States [1]. The incidence of breast cancer is about 1 in 8 women (13%) in the United States who will develop invasive breast cancer throughout their lifetime [2]. One common subtype of breast cancer is triple-negative breast cancer (TNBC), which has no progesterone receptor (PR), estrogen receptor (ER), or human epidermal growth factor 2 (HER2) [3]. TNBC is aggressive, metastatic, and highly invasive compared to other types of cancers, making treatment challenging. It constitutes 15% of diagnosed breast cancers and is found in African American and Caucasian women [4]. African American women diagnosed with TNBC are less likely to be treated with chemotherapy and surgery and are 28% more likely to die than Caucasian women with the same diagnosis [5]. It is well documented that there is an approximately twofold increased risk for TNBC in African Americans. African American women have more significant intratumor genetic heterogeneity and more aggressive tumor biology compared to Caucasian women, which can contribute to racial disparities in TNBC [6]. These disparities may be attributed to socioeconomic factors and tumor biology differences. It is well known that altering gene expression levels is associated with cellular growth, migration, and metastasis in breast tumors of African American and Caucasian women [7]. The genetic heterogeneity of TNBC tumors is higher in African Americans compared to Caucasian women. A study of 12 African American and Caucasian American matched BRCA patients analyzed 84 genes associated with tumor aggressiveness. Of the 84 expressed genes, 20 presented gene expression variation, showing that gene differences play a role in worsening clinical outcomes in African American women [8]. Specific somatic mutation gene analysis showed that for PIK3CA, 23% of the expression was established for African Americans and 34% demonstrated for Caucasians, and for TP53, 46% of the expression was shown for African Americans and 27% shown for Caucasians [9], confirming the genetic variability between races.

**Figure 1:**
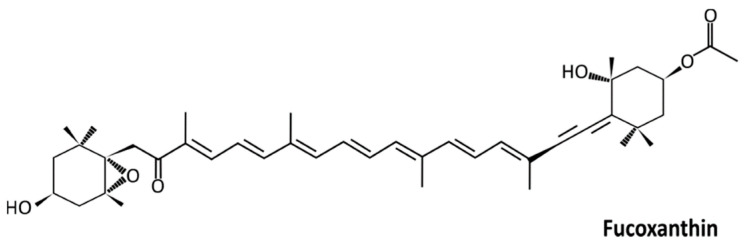
Chemical structure of fucoxanthin; C_42_H_58_O_6_; 658.91 g/mol.

In TNBC, the absence of progesterone receptors, estrogen receptors, and human epidermal growth factor receptor-2 restricts the treatment choices for TNBC. Moreover, conventional medication is not highly effective in treating TNBC, and developing effective therapeutic agents from natural bioactive compounds is a viable option. Natural compounds are usually less toxic and affordable and present different properties, including anticancer, anti-inflammatory, antiproliferative, antiangiogenic, and antioxidant properties [10]. For centuries, natural compounds have been used in Traditional Chinese Medicine [11]. Over the last decades, natural plant products have been used to develop new drugs. Microalgae, macroalgae, bacteria, plants, insects, and fungi are examples of photosynthetic organisms that produce carotenoids, a subfamily of tetraterpenoids or isoprenoids [12]. The natural orange-red food pigment of carotenoids is also found in many fruits and vegetables [13,14,15,16,17]. Macroalgae-derived fucoxanthin (3′-acetoxy-5, 6-epoxy-3,5′-dihydroxy-6′,7′-didehyro-5,6,7,8,5′,6′-hexahydro-β,β-carotene-8-one (Figure 1), a xanthophyll, is an orange-colored pigment that contributes to more than 10% of carotenoids in nature and is one of the most abundant carotenoids in the marine environment. It is isolated from brown algae such as *Undaria pinnatifida*, *Petalonia binghamiae*, *Sargassum siliquastrum*, *Laminaria ochotensis*, *Hijikia fusiformis*, and *Eisenia bicyclis* [18,19,20,21,22,23,24,25].

Fucoxanthin has shown various biological effects, including antiangiogenic, anti-tumoral, anticancer, antifungal, and antibacterial effects [25,26,27,28]. Previous studies have revealed fucoxanthin’s anticancer effects in gastric, bladder, skin, prostate, cervical, lung, and breast cancer [29,30,31,32,33,34,35]. Studies have also shown that fucoxanthin can induce G1 cell cycle arrest with apoptosis, induce autophagy at G2/M arrest, and reduce metastasis [36]. Fucoxanthin produces anticarcinogenic and antitumor effects by modulating the expressions of several signaling transduction pathways [37]. Moreover, fucoxanthin induces cell cycle arrest in human colon carcinoma at G0/G1 via the upregulation of p21WAF1/Cip1 [38].

In the gastrointestinal tract, fucoxanthin can be hydrolyzed into fucoxanthionol and broken down to amarociaxanthin A in the liver. There are many studies on fucoxanthin in the TNBC cells MDA-MB-231. Fucoxanthin and fucoxanthinol, its metabolite, have been found to induce anticancer effects in vitro and in vivo in MDA-MB-231 cells. In a Transwell migration assay, fucoxanthin inhibited migration on human lymphatic endothelial and MDA-MB-231 cells. In addition, fucoxanthin inhibited lymphangiogenesis induced by MDA-MB-231 cells. Fucoxanthin (100 μmol/L and 500 μmol/L) induced antiangiogenic effects in mice engrafted with MDA-MB-231 cells [39]. Moreover, fucoxanthionol (10 μΜ) increased apoptosis and lower cell viability in MDA-MB-231 cells. Also, Western blot analysis showed that fucoxanthinol decreased p100, p52, RelB, and NF-κB member p65 in MDA-MB-231 cells [40].

Many studies have examined fucoxanthin’s anticancer effect on the cell line MDA-MB-231. However, there is a lack of studies on the anticancer effect of fucoxanthin on MDA-MB-468 cells. Therefore, the current investigation is designed to investigate the differences in the anticancer effects of fucoxanthin on the two different TNBC cell lines, MDA-MB-231 and MDA-MB-468 cells, with further emphasis on exploring the molecular differences in the anticancer effects of fucoxanthin on these two genetically distinct TNBC lines.

## 2. Results

### 2.1. Fucoxanthin Inhibits Cell Viability in MDA-MB-231 and MDA-MB-468 Cell Lines

**Figure 2:**
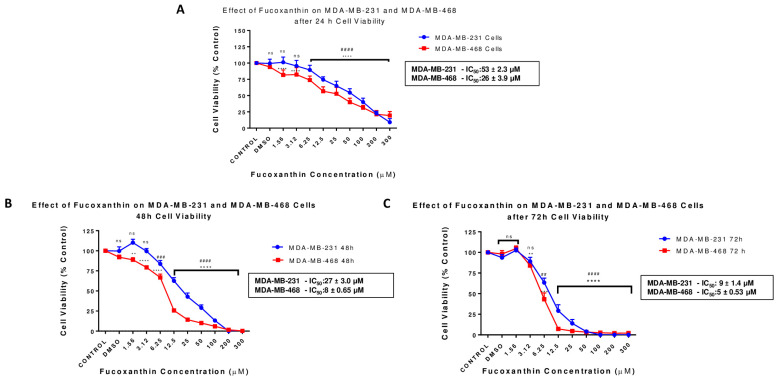
Effect of fucoxanthin on cell viability (Alamar blue) in MDA-MB-231 and MDA-MB-468 TNBC cells. Concentrations of fucoxanthin were tested in ranges from 1.56 to 300 µM. The control cells were treated with DMSO (<0.1%). Each experiment was performed three times with n = 5 at 5% CO_2_ and 37 °C. The cytotoxic effect was measured at 24 h (**A**), 48 h (**B**), and 72 h (**C**) treatment periods. The data are presented as the mean ± SEM. Statistically significant differences between control vs. treatments were evaluated by a one-way ANOVA, followed by Dunnett’s multiple comparison tests. ** *p* < 0.01, *** *p* < 0.001, **** *p* < 0.0001, ns = *p* > 0.05, ## *p* < 0.01, ### *p* < 0.001, #### *p* < 0.0001.

The effect of fucoxanthin on cell viability in TNBC was investigated in MDA-MB-231 and MDA-MB-468 cell lines after 24 h, 48 h, and 72 h treatment (Figure 2). Fucoxanthin showed a dose–response relationship in cell viability in both cell lines when compared to the DMSO-treated control cells; MDA-MB-468 cells were more sensitive to the compound, as revealed by the statistically significant decrease (*p* < 0.0001) in cell viability at all tested concentrations (from 1.56 µM to 300 µM). Meanwhile, this effect was found at higher concentrations in MDA-MB-231 cells (from 6.25 µM to 300 µM). The calculated IC50 validated the potential of the compound to induce a higher cytotoxic effect in MDA-MB-468 cells, giving IC50s values of 26 ± 3.88, 8 ± 0.65, and 5 ± 0.53 μM at the experimental periods (24, 48, and 72 h, respectively). However, the IC50 values in its counterpart, MDA-MB-231 cells, were 53 ± 2.30 μM, 27 ± 2.95, and 9 ± 2.95 μM at the same exposure periods (Figure 2A–C). The results show a time-dependent effect with higher potency in MDA-MB-468 cells at 48 h and 72 h compared to MDA-MB-231 cells.

### 2.2. Fucoxanthin Reduces Cell Growth in MDA-MB-231 and MDA-MB-468 Cell Lines

**Figure 3:**
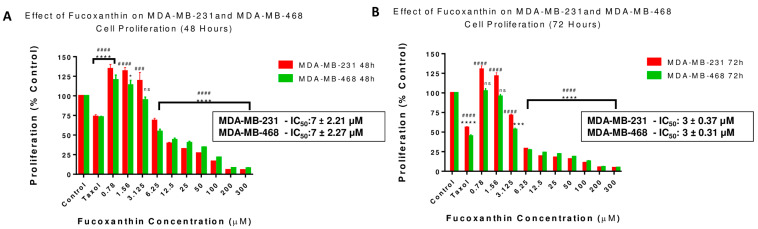
Effect of fucoxanthin on proliferation in MDA-MB-231 (**A**) and MDA-MB-468 (**B**) TNBC Cells at 48 h and 72 h. Fucoxanthin was tested at concentration ranges from 0.78 to 300 µM, and Taxol (1 µM) was used as a positive control. The cells were treated with DMSO (<0.1%). Each experiment was performed three times with n = 5 at 5% CO_2_ and 37 °C. The antiproliferative effect was measured at 48 and 72 h treatment in both cell lines (**A**,**B**). The data are presented as the mean ± SEM. Statistically significant differences between control vs. treatments were evaluated by a one-way ANOVA, followed by Dunnett’s multiple comparison tests. * *p* < 0.05, *** *p* < 0.001, **** *p* < 0.0001, ns = *p* > 0.05, ### *p* < 0.001, #### *p* < 0.0001.

Anti-proliferation assays were completed to establish the potency of fucoxanthin in inhibiting cell growth in both cell lines compared to the chemotherapeutic drug Taxol. Based on resazurin reduction, the antiproliferative effects of fucoxanthin were investigated by measuring cellular metabolic activity and their capacity to reduce resazurin after incubation at 48 and 72 h exposure times. The treatment concentration ranges for cells treated with fucoxanthin were 0.78 to 300 µM. The decrease in the cell proliferation rate was revealed in a dose–response behavior in both cell lines. Surprisingly, the obtained IC50 values did not show a significant difference at either the 48 h or 72 h exposure period (Figure 3A,B). Proliferation rates at 48 h exposure times in MDA-MB-231 (IC_50_: 7 ± 2.21) and MDA-MB-468 cells (IC_50_: 7 ± 2.27) (Figure 3A), and 72 h exposure times in MDA-MB-231 (IC_50_: 3 ± 0.37) and MDA-MB-468 cells (IC_50_: 3 ± 0.31) (Figure 3B) showed a significant decrease compared to the control groups beginning at the concentration of 0.78 µM. At 24 h, the lower concentration of 3.125 µM effectively reduced cell growth in both cell lines. More than 90% inhibition in cell proliferation was induced in both fucoxanthin-treated cell lines at the highest tested concentration of 300 µM (Figure 3A,B). Likewise, Taxol and fucoxanthin, after 48 and 72 h treatments, reduced TNBC cell growth and showed potency as an antiproliferative agent.

### 2.3. Fucoxanthin Effects on Cell Morphology

**Figure 4:**
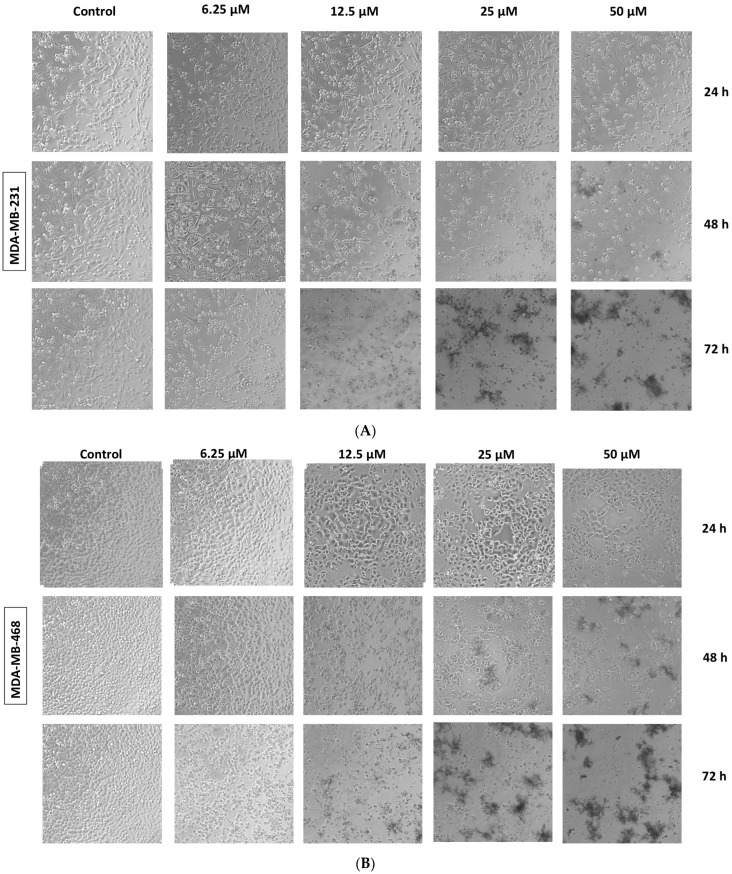
Morphological changes induced by fucoxanthin in MDA-MB-231 and MDA-MB-468 cells. Morphological changes induced by fucoxanthin (6.25–50 µM) in MDA-MB-231 (**A**) and MDA-MB-468 (**B**) cells were photographed at 24 h, 48 h, and 72 h. Cells were visualized using phase contrast and photographed at 40× magnification. Images were captured using the Olympus Cell Sens Standard Cytation5 cell Imaging reader (BioTek Instruments, Inc., Winooski, VT, USA).

A phase contrast microscope was used to analyze the effects of fucoxanthin on the cellular morphology of MDA-MB-231 and MDA-MB-468 cell lines at 24 h, 48 h, and 72 h. Cells were photographed at 40× magnification. Fucoxanthin induced noticeable morphological changes in both cell lines at concentrations ranging from 6.25 to 50 µM and at 24–72 h exposure periods. In the MDA-MB-231 cells (Figure 4A), the control group cells that were treated with DMSO (<0.1%) showed intact, long, and thin spindle-shaped. At 24 h, beginning at a concentration of 25 μM, the cells shifted from a spindle to a small spherical shape and showed lower density. At 48 h and 72 h, fucoxanthin in concentrations from 12.5 to 50 μM caused the cells to appear with smaller rounded shapes and lower density. In the MDA-MB-468 cells (Figure 4B), the control group showed grape-like aggregate-shaped cellular structures. Similarly, as the MDA-MB-231 cells, after 48 and 72 h, fucoxanthin concentrations from 12.5 to 50 μM displayed further shrunken to smaller rounded cellular shapes and lower cell density.

### 2.4. Fucoxanthin Inductive Effects of Apoptosis in MDA-MB-231 and MDA-MB-468 Cells

**Figure 5:**
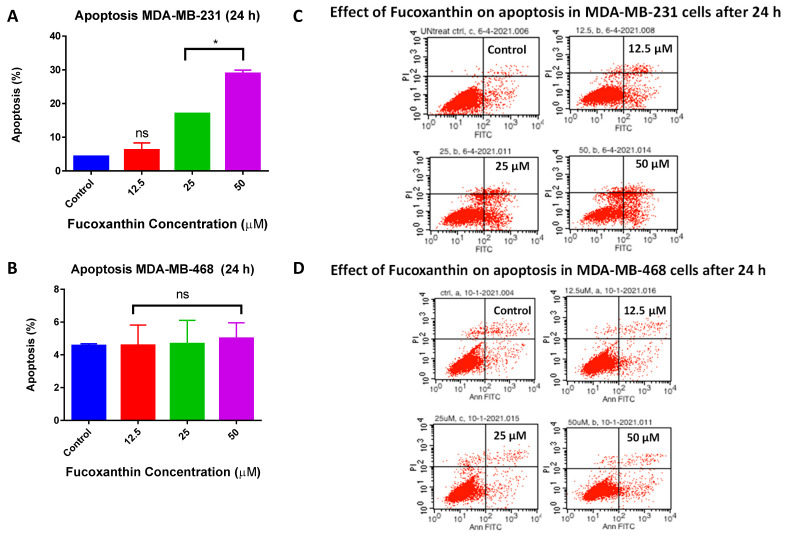
Ability of fucoxanthin to induce apoptosis in MDA-MB-231 (**A**,**B**) and MDA-MB-468 TNBC cells (**C**,**D**). In six-well plates, cells were treated with DMSO (control) and selected concentrations of fucoxanthin ranging from 12.5 to 50 µM. At 24 h, the apoptotic effect of fucoxanthin was quantified using a FACSCalibur Flow cytometer (Becton Dickinson, San Jose, CA, USA) and analyzed by the CELLQuest software. The lower left quadrant represents alive cells, lower right quadrant represents early apoptotic cells, upper right quadrant represents late apoptotic cells, and upper left quadrant represents necrotic cells. The data are presented as the mean ± SEM, n = 3. Statistically significant differences between control vs. treatments were evaluated by a one-way ANOVA, followed by Dunnett’s multiple comparison tests. * *p* < 0.05 and ns = *p* > 0.05.

An Annexin V-FITC apoptosis assay was used to examine the inductive effects of fucoxanthin on apoptosis in MDA-MB-231 and MDA-MB-468 cells. The apoptotic effects of fucoxanthin were investigated at 24 h and quantified using a flow cytometer. Annexin V detects and binds phosphatidylserines, which flip to the outer plasma membrane of apoptotic cells, while DNA binding dye propidium iodide (PI) is used to detect necrotic or late apoptotic cells. Fucoxanthin-treated MDA-MB-231 cells showed a statistically significant increase (* *p* < 0.05) of apoptotic cells compared to the control group. Fucoxanthin induced apoptosis (16.88% and 25.40%) at concentrations of 25 µM and 50 µM, respectively; meanwhile, there was no effect on the induction of apoptosis at the lowest tested concentration of 12.5 μM compared to the control group (Figure 5A,B). In MDA-MB-468 cells, fucoxanthin had no apoptotic effect at any of the tested concentrations compared to the control group (Figure 5C,D). The flow cytometry analysis shows quadrants containing alive cells (lower left), early apoptotic cells (lower right), late apoptotic cells (upper right), and necrotic cells (upper left).

### 2.5. Fucoxanthin Induces Cell Cycle Arrest in MDA-MB-231 and MDA-MB-468 Cells

**Figure 6:**
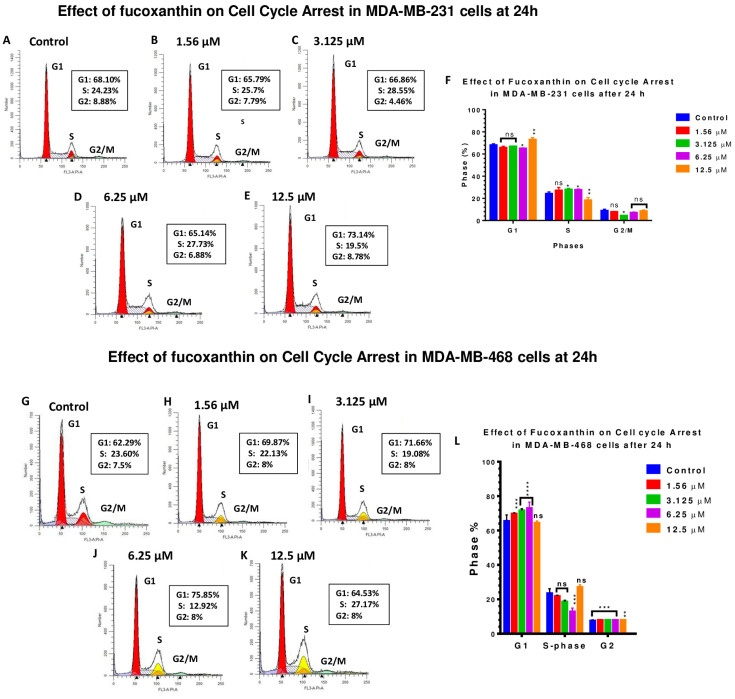
Potential of fucoxanthin to induce cell cycle arrest in MDA-MB-231 (**A**–**F**) and MDA-MB-468 TNBC cells (**G**–**L**). Cells were treated with DMSO (control) and selected concentrations of fucoxanthin ranging from 1.56 to 12.5 µM. At 24 h, the ability of fucoxanthin to induce cell cycle arrest was quantified using a FACSCalibur Flow cytometer (Becton Dickinson, San Jose, CA, USA). The data are presented as the mean ± SEM. Statistically significant differences between control vs. treatments were evaluated by a one-way ANOVA, followed by Dunnett’s multiple comparison tests. * *p* < 0.05, ** *p* < 0.01, *** *p* < 0.001, **** *p* < 0.0001, ns = *p* > 0.05.

The potential of fucoxanthin to induce cell cycle arrest was investigated in MDA-MB-231 (Figure 6A–F) and MDA-MB-468 (Figure 6G–L) cell lines at 24 h. In both cell lines, the compound was tested at concentration ranges from 1.56 to 12.5 μΜ. A Sony SH800 cell sorter and flow cytometry were used to analyze cells’ distribution among different cell cycle phases. In MDA-MB-231 cells, fucoxanthin significantly (*p* < 0.01) induced cell cycle arrest at G1-phase at 12.5 μM (73.14%, Figure 6E), compared to 68.10% in DMSO-treated control cells (Figure 6A). This increase was accompanied by a significant 5% decrease (*p* < 0.01) of S-phase. This pattern of MDA-MB-231 response was reversed at lower concentrations of the compound compared with the control cells (Figure 6A–D,F). In parallel, the significant decrease in cells at the G2 phase was only significant (*p* < 0.05) at 3.125 μM. In the MDA-MB-468 cells, a gradual and significant increase in cells at the G1 phase was found at concentrations of 1.56 μM–6.25 μM (*p* < 0.001–*p* < 0.0001) of fucoxanthin (69.87–80.25%) (Figure 6H–K), while the percentage of control cells at the G1 phase was 62.29% (Figure 6G). In return, a significant decrease (*p* < 0.001) in the S-phase was only found at 6.25 μΜ of the compound. Interestingly, a minor, but significant, increase in G2 (*p* < 0.01–*p* < 0.001) was induced by fucoxanthin at all tested concentrations. In comparison, a ~5% increase in MDA-MB-231 cells at the G1 phase was measured at 12.5 μM of fucoxanthin. Meanwhile, an 18% increase in cells arrested at the same phase was obtained at 6.25 μM of the compound in MDA-MB-468 cells. In both cell lines, this increase in cells arrested at the G1 phase was parallel with a reduction in the S-phase. Meanwhile, a less than 5% reduction was measured in MDA-MB-231 cells at 12.5 μM, and a 54.74% decrease in MDA-MB-468 cells was detected at a lower concentration (6.25 μM) compared to the control cells.

### 2.6. Fucoxanthin Inhibited Cellular Migration in MDA-MB-231 and MDA-MB-468 Cells

**Figure 7:**
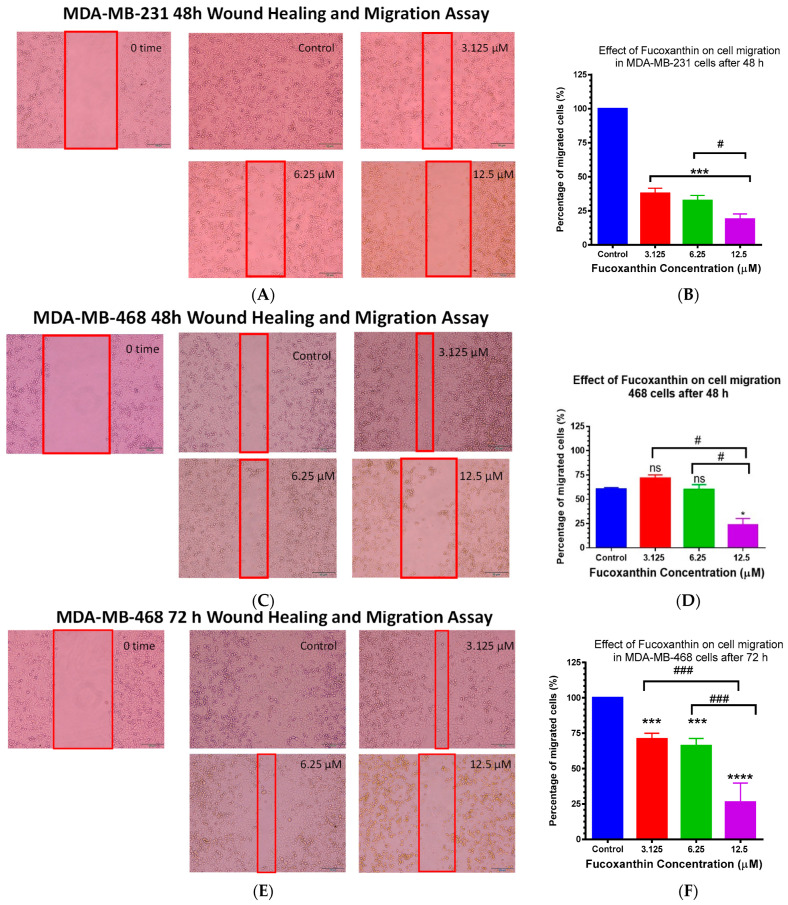
Effect of fucoxanthin on cell migration in MDA-MB-231 (**A**,**B**) and MDA-MB-468 cells (**C**,**D**) and (**E**,**F**) using a culture inserts two-well (Ibidi) migration assay after 48 h and 72 h, respectively. In six-well plates, MDA-MB-231 and MDA-MB-468 cells with identical densities were seeded and incubated overnight. On the next day, all inserts were removed, and the cells were treated with selected concentrations of fucoxanthin (3.125 μM, 6.25 µM, and 12.5 µM) and DMSO (control). Images were captured at 0 h exposure time using an Olympus cell Sens Standard. All plates were monitored until the control group gaps were closed. The control group gaps were sealed at 48 h exposure period (MDA-MB-231 cells) and 72 h exposure period (MDA-MB-468 cells). The images were captured to measure the width of the gaps. Data are the average of three independent studies (n = 3). Statistically significant differences between control vs. different fucoxanthin concentrations (*) were analyzed using one-way ANOVA followed by Dunnett’s multiple comparison tests. *t*-test was used to calculate the statistically significant differences between the three different treatment groups (3.125 vs. 6.25), (3.125 vs. 12.5), and (6.25 vs. 12.5). * *p* < 0.05, *** *p* < 0.001, **** *p* < 0.0001, # *p* < 0.05, ### *p* < 0.001, and are statistically significant, ns = *p* > 0.05.

A culture inserts two-well (Ibidi) migration assay was used to analyze the inhibitory effect of fucoxanthin on migration in MDA-MB-231 and MDA-MB-468 cell lines. As an endpoint, the gap for the control wells was closed after 48 h in MDA-MB-231 cells. Meanwhile, it took 72 h in its counterpart, MDA-MB-468 cells. A significant (*p* < 0.001) and gradual decrease in cell migration was found in fucoxanthin-treated TNBC cells at the three tested concentrations (3.125, 6.25, and 12.5 μM). In both cell lines, more than 70% inhibition of the migrated cells was found at the highest treated concentration (12.5 μM). However, this effect was found at 48 h exposure in MDA-MB-231 and MDA-MB-468 cells (Figure 7A/B) and (Figure 7C/D); meanwhile, it took a longer time (72 h) in MDA-MB-468 cells (Figure 7E/F). Compared to the control cells, significant inhibition of migrated MDA-MB-468 cells was only found at 12.5 μM after 48 h exposure (*p* < 0.05) (Figure 7B). At the same time, the same concentration has a significant relation (*p* < 0.05) when compared with either 3.125 or 6.25 uM. In MDA-MB-468 cells after 72 h, there was also a statistically significant decrease in the percentage of migrated cells with increased concentrations (3.125–12.5 μM) (*p* < 0.0001–*p* < 0.001) compared to the control. *t*-tests showed statistical significance (### *p* < 0.001) comparing 12.5 μM vs. 6.25 μM or 3.125 μM. Hence, our results showed the effect of fucoxanthin on cell migration in both cell lines.

### 2.7. Fucoxanthin Inhibited the Protein Expression of VEGF-A in TNF-α-Stimulated MDA-MB-231 Cells

**Figure 8:**
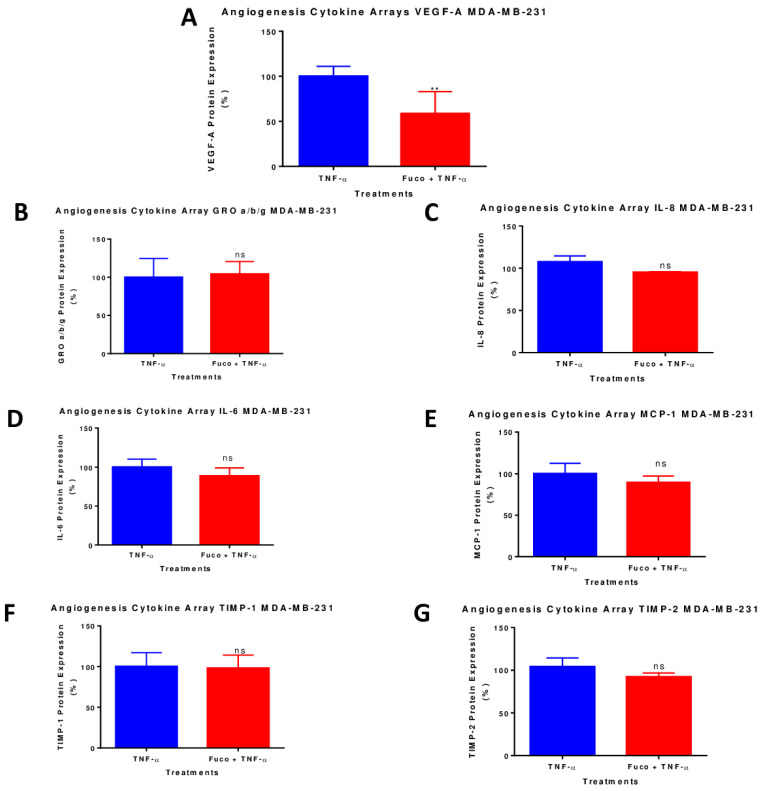
Normalized protein expression of VEGF-A (**A**), GRO a/b/g (**B**), IL-8 (**C**), IL-6 (**D**), MCP-1 (**E**), TIMP-1 (**F**), and TIMP-2 (**G**) in MDA-MB-231 TNBC cells. Data show normalized protein expressions from the cytokine arrays calculated based on the positive controls found in the corners of each membrane using RAYBIO^®^ANALYSIS software RayBiotech (Nocross, GA, USA). Data are expressed as mean ± SEM n = 3, representing two treatments: TNF-α (50 ng/mL) and fucoxanthin (6.25 µM) + TNF-α (50 ng/mL). Statistically significant differences between TNF-α vs. fucoxanthin + TNF-α were previously evaluated by an unpaired *t*-test. ** *p* < 0.01 and ns = *p* > 0.05.

**Figure 9:**
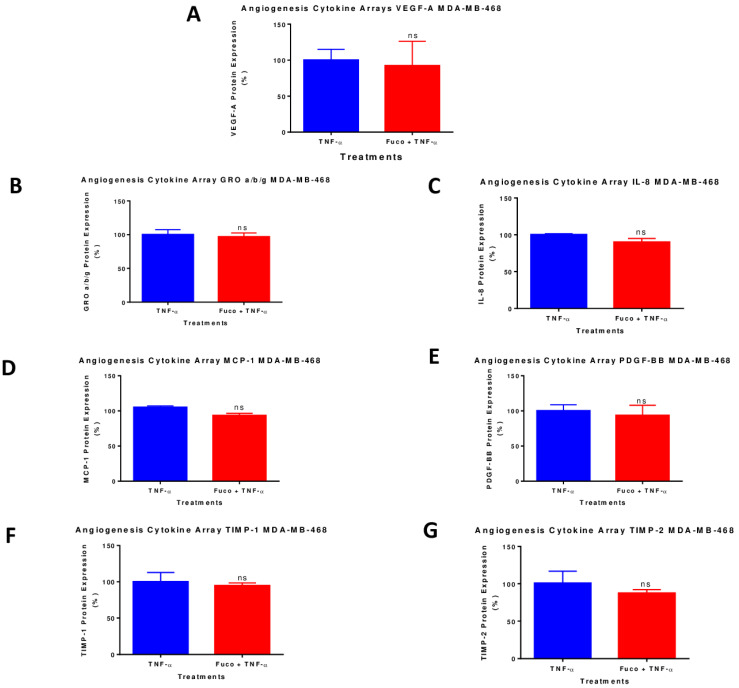
Normalized protein expression of VEGF-A (**A**), GRO a/b/g (**B**), IL-8 (**C**), MCP-1 (**D**), PDGF-BB (**E**), TIMP-1 (**F**), and TIMP-2 (**G**) in MDA-MB-468 TNBC cells. Data show normalized protein expressions from the cytokine arrays calculated based on the positive controls found in the corners of each membrane using RAYBIO^®^ANALYSIS software (RayBiotech). Data are expressed as mean ± SEM n = 3, representing two treatments: TNF-α (50 ng/mL) and fucoxanthin (6.25 µM) + TNF-α (50 ng/mL). Statistically significant differences between TNF-α vs. fucoxanthin + TNF-α were evaluated by an unpaired *t*-test and ns = *p* > 0.05.

A semi-quantitative analysis using human angiogenesis antibody arrays was used to evaluate the relationship between the anticancer effects of fucoxanthin and its inhibitory effect on TNF-α-activated proinflammatory cytokines Our results show that TNF-α induced the upregulation of seven cytokines in MDA-MB-231 cells: growth-regulated proteins (GRO α/β/γ), Interleukin-6 (IL-6), tissue inhibitor of metalloproteinases-1 (TIMP-1), Interleukin-8 (IL-8), C-X-C motif chemokine ligand 8 (CXCL8), tissue inhibitor of metalloproteinases 2 (TIMP-2), monocyte chemoattractant protein-1 (MCP-1) chemokine (C-C motif) Ligand 2 (CCL2), and VEGF-A. After treatment with TNF-α and fucoxanthin, only the expression of VEGF-A was inhibited in the MDA-MB-231 cells (Figure 8A); fucoxanthin did not affect the expression of the other proteins upregulated by TNF-α (Figure 8B–G). In the MDA-MB-468 cells, our results show that TNF-α induced the upregulation of seven cytokines: growth-regulated proteins (GRO α/β/γ), tissue inhibitor of metalloproteinases-1 (TIMP-1), Interleukin-8 (IL-8), C-X-C motif chemokine ligand 8 (CXCL8), tissue inhibitor of metalloproteinases 2 (TIMP-2), monocyte chemoattractant protein-1 (MCP-1) chemokine (C-C motif) Ligand 2 (CCL2), platelet-derived growth factor-BB (PDGF-BB), and VEGF-A. Fucoxanthin treatment caused no change in the expression of VEGF-A (Figure 9A–G) or any other protein that was induced by TNF-α in the MDA-MB-468 cells, showing a difference in the response of these cells towards the compound.

### 2.8. Fucoxanthin Inhibited TNF-α-Induced VEGF-A and VEGF-C mRNA Expression in MDA-MB-231 Cells

**Figure 10:**
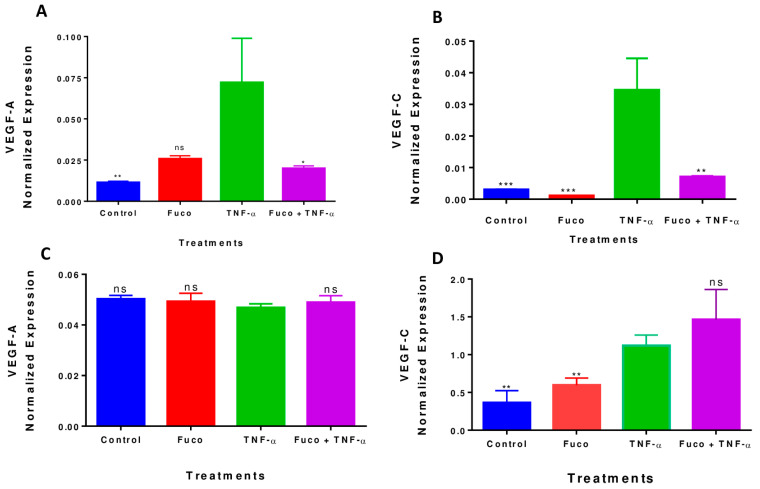
Effect of fucoxanthin on VEGF-A and VEGF-C expressions in MDA-MB-231 and MDA-MB-468 Cells. The effect of fucoxanthin in VEGF-A (**A**,**C**) and VEGF-C (**B**,**D**) protein expression in MDA-MB-231 and MDA-MB-468 cells. Each point represents the mean ± SEM, representing four treatments: control (cells + DMSO), fucoxanthin (6.25 µM), TNF-α (50 ng/mL), and fucoxanthin (6.25 µM) + TNF-α (50 ng/mL). A one-way ANOVA and Dunnett’s multiple comparison tests statistically evaluated the differences between TNF-α vs. fucoxanthin + TNF-α. * *p* < 0.05, ** *p* < 0.01, *** *p* < 0.001, *p* < 0.05, ns = *p* > 0.05.

RT-PCR was used to investigate the effect of fucoxanthin in VEGF-A and VEGF-C mRNA expressions in the MDA-MB-231 cell line. TNF-α treatment induced VEGF-A (Figure 10A) and VEGF-C (Figure 10B) mRNA expression in MDA-MB-231 cells compared to the controls. However, when cells were treated with TNF-α and fucoxanthin, VEGF-A and VEGF-C expressions were significantly reduced (* *p* < 0.05 and ** *p* < 0.01, respectively) compared to the control. In the MDA-MB-468 cells, fucoxanthin neither inhibits the expression of VEGF-A nor VEGF-C (Figure 10C,D). Gene expression for VEGF-A and VEGF-C was normalized using GAPDH, and the normalization was calculated automatically using BIO-RAD CFX-6 software (Bio-Rad, Hercules, CA, USA). These results confirm the results from the antibody arrays and show that fucoxanthin is effective in reducing VEGF-A and VEGF-C mRNA expression in MDA-MB-231 cells, but not in MDA-MB-468 cells.

## 3. Discussion

The potent effects of marine carotenoids against various cancer cell lines have been mediated by transcription factors, cell proliferation, metastasis, and angiogenesis [41]. Seaweed contains important bioactive compounds with beneficial effects such as anti-inflammatory, antioxidant, antidiabetic, anti-obesity, and anticancer activities [42]. Fucoxanthin, a xanthophyll subset of carotenoids, seaweed, and marine macroalgae, is potentially an anticancer therapeutic for TNBC [43]. Previous studies indicated fucoxanthin’s potential to inhibit proliferation and angiogenesis in cancer cell lines [44]. It has also been reported that fucoxanthin induces apoptosis and cell cycle growth arrest in several cancer cell lines, such as breast, gastric, lung, bladder, and prostate cancer cell lines (detailed in our previous paper) [45,46]. In human bladder cancer T24 cells and human colon cancer cells, fucoxanthin induced cell cycle growth arrest at the G_0_/G_1_ phase of the cell cycle [36]. Fucoxanthin has been shown to downregulate the mRNA expression of VEGF-C in MDA-MB-231 cells [47].

Previous studies have shown the cytotoxic, antiproliferative, apoptotic, and cell cycle arrest effects of fucoxanthin in MDA-MB-231 TNBC cells [35]; however, no studies on the effects of fucoxanthin on MDA-MB-468 TNBC cells have been reported. The difference in the genetic tumor profiles of patients has been proposed to contribute to the disparity in TNBC cells [48]. Gene alteration also contributes to differences in clinical outcomes between both African American and Caucasian American women with TNBC. VEGF is known to be overexpressed in African American compared to Caucasian American breast cancer patients [49]. Cytokines such as IL-6 have been reported to be related to higher aggressiveness in African American women with TNBC [50]. A study between both cell lines showed that from 84 genes, 20 were differentially expressed [51]. The Cancer Genome Atlas (TCGA) was used to perform a comprehensive study between both populations, and 674 genes were found to have variation. The current study demonstrates the inhibitory anticancer effects of fucoxanthin in genetically different MDA-MB-231 and MDA-MB-468 cell lines.

Based on the results of this study, fucoxanthin showed cytotoxic and antiproliferative effects in both cell lines while displaying higher potency at lower concentrations in MDA-MB-468 cells compared to MDA-MB-231 cells. An increase in fucoxanthin concentration caused a decrease in cell viability after 24 h, 48 h, and 72 h treatment periods, thus showing a dose and time response in both cell lines. Anti-proliferation assays were completed to establish the potency of fucoxanthin in the inhibition of cell growth in both cell lines, compared to the chemotherapeutic drug Taxol. After 48 h and 72 h of fucoxanthin treatment, proliferation assay studies showed a similar response in both cell lines. It is well known that morphological changes occur in cells undergoing apoptosis. Apoptosis is the process of programmed cell death and may be induced by natural products for breast cancer prevention [52]. In this study, in both cell lines at 48 h and 72 h, fucoxanthin (25–50 μM) induced morphological changes in the cells. The cells showed morphological changes such as cell shrinkage, loss of adhesion, DNA fragmentation, nuclear fragmentation, and blebbing, which are characteristics of cells undergoing apoptosis [53].

Cell cycle arrest and apoptosis must be balanced to maintain homeostasis [54]. Both apoptosis and cellular proliferation are linked by cell cycle regulators and apoptotic stimulation, which affects both processes [55]. Apoptosis occurs when cell cycle checkpoints are interrupted. Proteins involved in apoptosis and cell cycle pathways are known to induce cellular death, cell cycle arrest, or cell proliferation, thereby contributing to tumor progression. Apoptosis cell features include DNA fragmentation, nuclear fragmentation, chromatin aggregation, mRNA decay, and the formation of apoptotic bodies [56]. The lack of apoptosis can lead to uncontrolled cellular proliferation, and excessive apoptosis leads to atrophy. In normal cells, there is a balance between apoptosis and proliferation. However, a dysregulated balance between proliferation and apoptosis activates antiapoptotic and proapoptotic signaling pathways [57]. Apoptosis evasion is an important mechanism for metastatic cancer cells [58]. The induction of apoptosis is a profound mechanism that natural anticancer agents trigger. Indeed, natural compounds isolated from plants are used for breast cancer treatment through apoptosis induction. [59]. In this study, fucoxanthin’s apoptotic effects were performed in both cell lines. The results showed that fucoxanthin (25–50 μM) induced apoptosis in MDA-MB-231, whereas in MDA-MB-468 cells, there was no apoptotic effect, showing a difference in the response of these cells towards fucoxanthin. The data revealed that there was a statistically significant increase (* *p* < 0.05) in apoptotic cells and 16.98% to 28.89% in induced apoptosis by fucoxanthin treatment in MDA-MB-231 cells after 24 h. Previous studies have reported fucoxanthin-induced apoptosis in MDA-MB-231 and MCF-7 at 10 μΜ [40]. Studies have shown that apoptosis is the opposite of cell growth, and for a tumor to effectively grow, cells must hijack cell growth pathways and evade apoptosis [52].

Our cell viability results showed that fucoxanthin was more effective at lower concentrations in the MDA-MB-468 cells vs. MDA-MB-231 cells, as indicated by apoptosis assay. Moreover, cell proliferation is a process that involves an increase in cell number and defines the balance between cell loss and cell divisions [60]. During the division of cells, the cell cycle ensures the integrity of checkpoints and assesses the integrity of dividing cells. The dysregulation of the cell cycle participates in cancer development and progression [61]. The cell cycle is a series of synchronized events of four phases, including the G1, S, G2, and M phases, which advance to cellular division. A steady cell cycle arrest indicates the incapacity of the cell to continuously divide, which is one of the features of senescent cells [62]. The transition from the G1 to the S phase of the cell cycle is vital for controlling cell proliferation and promoting carcinogenesis [63]. The G2/M checkpoint prevents cells from entering mitosis when DNA is damaged, and it has been shown that after the G2/M phase, breast cancer cell lines exhibit apoptosis [64]. Cell cycle arrest at G1 and S phases can be utilized for cancer therapy. Cell cycle arrest and apoptosis in the G1 period signify distinctive ways of cellular growth arrest that result in a cell’s incapability to move into the S phase [65]. Studies showed that fucoxanthin can block the S phase of the cell cycle by inhibiting proliferation [21]. Fucoxanthin has been found to induce cell cycle arrest in human colon carcinoma cells through the upregulation of p21WAF1/Cip1 [38].

Based on this investigation, fucoxanthin induced cell cycle arrest at both the G1 phase and the S phase in MDA-MB-231 and MDA-MB-468 cells. The results also showed that fucoxanthin induced cell cycle arrest at the G1 phase in MDA-MB-231 cells at 12.5 μM (73.14%) compared to control cells (68.10%), and in MDA-MB-468 cells between 1.56 and 12.5 μΜ (64.53–80.25%) compared to control cells (62.24%). Fucoxanthin induced cell cycle arrest at the S-phase in MDA-MB-231 cells at higher percentages between concentrations of 1.56 and 6.25 μM (25.7–28.55%) compared to control cells (24.23%). Cell cycle arrest was induced by fucoxanthin at the S-phase in MDA-MB-468 cells at a concentration of 12.5 μM (27.71%) compared to control cells (23.60%). Lastly, fucoxanthin induced cell cycle arrest at the G2 phase in MDA-MB-468 cells at concentrations of 1.56–12.5 μM (8%) compared to control cells (7.5%). Therefore, the results show that fucoxanthin modulates the cell cycle of the selected TNBC cells, but the concentrations may vary according to the cell line tested.

Chronic inflammation initiated by the tumor microenvironment is an alternative process that drives cancer initiation, progression, proliferation, metastasis, and chemotherapeutic resistance [66]. Inflammation plays a role in tumor progression, and the tumor microenvironment employs several inflammatory cytokines that are essential for tumor development and inflammatory promotion in TNBC [67]. TNF-α is an inflammatory cytokine highly expressed in breast tumors and has been shown to induce several cellular processes, such as cell survival, cell proliferation, cell cycle, mitosis, and metastasis [68]. In this study, the effect of fucoxanthin on angiogenesis was investigated by stimulating the TNBC cells with TNF-α. Angiogenesis is a new blood vessel growth mechanism that provides tumors with the nutrients and proteins needed to grow and survive [69]. This process is vital to developing metastasis and tumor growth sustained by angiogenic growth factors, such as VEGF [70]. The data showed a downregulation of VEGF-A protein expression in MDA-MB-231 cells, but no change in VEGF-A in MDA-MB-468 cells. There was no change in GRO a/b/g, IL-8, MCP-1, TIMP-1, and TIMP-2 expression in both cell lines. In addition, no change in the expression of IL-6 in MDA-MB-231 and PDGF-BB in MDA-MB-468 cells was shown. We also investigated fucoxanthin’s antiangiogenic effect on VEGF-A and VEGF-C mRNA expression in TNF-α-stimulated MDA-MB-231 cells and MDA-MB-468 cells. The results showed that both VEGF-A and VEGF-C expression had a significant decrease in MDA-MB-231 cells and no expression change in MDA-MB-468 cells. VEGF is a major angiogenic factor that supports metastatic invasion and angiogenesis while increasing vascular permeability [71]. Tumorigenesis is associated with pro-angiogenic VEGF, secreted from tumor cells, and promotes proliferation, cell growth, angiogenesis, and metastasis [72]. The release of VEGF through activating pathways such as NF-κB, MAPK, or JNK promotes angiogenesis [73,74,75]. The therapeutic use of fucoxanthin in cancer treatment has decreased VEGF expression [43]. Fucoxanthin has been shown to reduce NF-κB, phospho-Akt, phospho-phosphoinositide 3 kinase (PI3K), VEGF-C, and VEGFR-3 expression levels in human lymphatic endothelial cells (HLEC) [39]. Poor clinical outcomes in breast cancer tumors are associated with higher VEGF expression levels [76].

Therefore, the results of the present study address a possible variation in the immune response of the two types of TNBC cells selected for this investigation, which may be affected by VEGF expression and contribute to the increased risk of breast cancer development and progression. Studies have shown genetic variations between highly invasive and metastatic-natured MDA-MB-231 and MDA-MB-468 cells [77]. Caucasian American TNBC cells display higher expression levels of genes associated with cell motility and invasion. African American TNBC cells show higher expression levels of genes associated with differentiation, proliferation, and cellular adhesion [78,79]. In our studies, fucoxanthin inhibited cell viability, proliferation, cellular migration, and induced cell cycle arrest in both MDA-MB-231 and MDA-MB-468 cell lines. However, fucoxanthin was shown to induce apoptosis and downregulated genes and proteins associated with angiogenesis in MDA-MB-231 cells only. The MDA-MB-231 cells were more susceptible to the anticancer effects of fucoxanthin, showing that the MDA-MB-468 cells are less responsive to fucoxanthin treatment and have a more aggressive nature. The literature indicates that the PI3K/AKT signaling pathway plays several roles in regulating cellular processes such as metastasis, proliferation, cell cycle arrest, apoptosis, migration, and angiogenesis [80]. Our future studies will focus on the molecular mechanism of fucoxanthin involving the PI3K-AKT signaling pathway to elucidate why the compound is more effective in MDA-MB-231 than MDA-MB-468 cells.

## 4. Materials and Methods

### 4.1. Cell Lines, Chemicals, and Reagents

MDA-MB-231 (Caucasian American) and MDA-MB-468 (African American) TNBC cells were purchased from the American Type Culture Collection (ATCC). Fetal bovine serum heat-inactivated (FBS-HI), Dulbecco’s modified Eagle’s medium (DMEM) high glucose, penicillin/streptomycin, and phosphate-buffered saline (PBS) were obtained from Genesee Scientific (San Diego, CA, USA). Alamar Blue^®^, fucoxanthin (95% purity), and dimethyl sulfoxide (DMSO) were purchased from Sigma-Aldrich Co. (St. Louis, MO, USA). Human Angiogenesis Antibody Array (Cat# AAH-ANG-1000-4) and TNF-alpha (Cat# ELH-TNFα) were purchased from RayBiotech (Norcross, GA, USA). PCR arrays, SYBR Green, and iScript advanced reverse transcriptase kit were purchased from Bio-Rad (Hercules, CA, USA), and the propidium iodide DNA staining kit was purchased from Abcam (Boston, MA, USA). Wes kit and reagents were attained from ProteinSimple (San Jose, CA, USA), and antibodies were obtained from Cell Signaling Technology (Danvers, MA, USA) and ThermoFisher (Waltham, MA, USA).

#### Cell Culture

MDA-MB-231 and MDA-MB-468 TNBC cells were grown in DMEM complemented with 10% FBS-HI and 1% penicillin (100 U/mL)/streptomycin (0.1 mg/mL) and incubated at 37 °C and 5% CO_2_. Before use in each assay, the cells were subcultured in T-75 flasks and grown to 90% confluency. All assays were established using DMEM medium supplemented with 2.5% heat-inactivated FBS, with no penicillin/streptomycin.

### 4.2. Cell Viability

Cells were seeded in 96-well plates at a density of 3 × 10^4^ cells/100 µL/well and incubated overnight in experimental media to attach. After overnight incubation, the cells were exposed to either media only, media + DMSO, or various concentrations of fucoxanthin (1.56–300 µM) for another 24 h, 48 h, and 72 h. Fucoxanthin was dissolved in DMSO before dilution in media, and the final concentration of DMSO did not exceed 0.1%. A volume of 100 µL of each treatment was added to the plated cells and incubated according to the predesigned exposure period. Alamar Blue^®^ (Resazurin) reagent assessed MDA-MB-231 and MDA-MB-468 cell viability. The volume of 20 µL of Alamar Blue^®^ solution (0.5 mg/mL) was added to each well and incubated for 4 h. Fluorescence of excitation/emission at 550/580 nm wavelengths was measured using an Infinite M200 microplate reader (Tecan Trading AG (Morrisville, NC, USA). Fluorescence changes were observed as viable cells reduced resazurin to resorufin. The number of living cells in the sample was proportional to the fluorescent signal, and the data were expressed as a percentage of live untreated controls.

### 4.3. Cell Proliferation

Cells were seeded in 96-well plates at a density of 5 × 10^3^ cells/well and incubated overnight in experimental media to attach. After 24 h, the plated cells were treated in another 100 µL of the experimental media as follows: control (media + DMSO), positive control (Media Taxol 1 µM), or various concentrations of fucoxanthin (0.78–300 µM). Fucoxanthin was dissolved in DMSO before dilution in media, and the final concentration of DMSO did not exceed 0.1%. The effect of fucoxanthin was assessed at 48 and 72 h incubation periods. Alamar Blue^®^ (Resazurin) assay measured MDA-MB-231 and MDA-MB-468 cell proliferation, as previously mentioned in the cell viability assay (Section 4.2).

### 4.4. Apoptosis Assay

An Annexin V-FITC Apoptosis assay kit from RayBiotech was used to determine the ability of fucoxanthin to induce apoptosis in MDA-MB-231 and MDA-MB-468 cells. Cells were seeded in six-well plates at a concentration of 5 × 10^5^ cells/well and incubated overnight. The cells were treated with various concentrations of fucoxanthin (12.5–50 µM). Control cells were treated with DMSO at a concentration of <0.1%. Treated cells and controls in each well were harvested, pelleted, and washed with PBS after 24 h of incubation. Based on the manufacturer’s protocol, the cell pellets were resuspended in 500 µL of 1X Annexin-V binding buffer, and then labeled with 5 μL of Annexin V-FITC and 5μL of propidium iodide. Lastly, within 5–10 min, the apoptotic effect of fucoxanthin was quantified by FACSCalibur Flow cytometer (Becton Dickinson, San Jose, CA, USA). Each sample of 1 × 10^4^ cells was examined and analyzed by the CELLQuest software Pro/Version 5.1 (BD Biosciences, San Jose, CA, USA).

### 4.5. Cell Cycle Arrest Analysis

Flow cytometry analysis using a propidium iodide DNA staining kit (Abcam USA) was employed to assess the impact of fucoxanthin on cell cycle progression in MDA-MB-231 and MDA-MB-468 cells. Cells were seeded at 1 × 10^6^ cells/flask and incubated overnight. The cells were treated with various concentrations of fucoxanthin (1.56–12.5 µM) and incubated for another 24 h. Meanwhile, the control cells were treated with DMSO at a concentration of <0.1%. Cells from each flask were harvested, pelleted, and washed with cold PBS. Cells were fixed in cold HPLC ethanol and vortexed; all clumps were removed. Before analysis, cells were centrifuged, and ethanol/PBS was aspirated. Cells were then treated with 250 μL of a cocktail (PBS, PI, and ribonuclease) and incubated for 30 min. Lastly, cell cycle phases were quantified using the FACSCalibur Flow cytometer (Becton Dickinson, San Jose, CA, USA). Each sample of 1 × 10^4^ cells was examined and analyzed using the CELLQuest software Pro/Version 5.1 (BD Biosciences, San Jose, CA, USA).

### 4.6. Wound Healing Assay

The culture inserts two-well (Ibidi) was used to establish the migration assay. Two-well culture inserts were carefully placed in the middle of the six-well plates. MDA-MB-231 and MDA-MB-468 cells were seeded into each insert at a 3.5 × 10^4^/70 µL/well density and incubated overnight in complete media. After 24 h, the inserts were removed gently, and each well was washed with PBS to remove any suspended cells. Control-designated wells were exposed to DMSO. Meanwhile, other wells were incubated to selected fucoxanthin concentrations (3.125, 6.25, and 12.5 µM). Snapshot pictures of cell migration were taken at 0 h in both cell lines, at 48 h in MDA-MB-231 cells, and up to 72 h in MDA-MB-468 cells for each concentration. The images of the wells were captured by the Olympus IX73 microscope, visualized using OLYMPUS cell Sens Standard, and analyzed using PowerPoint. The following equation was used to calculate the percentage of migrated cells:

$$\frac{A2}{A1\;}\;\left(Treated\;cells\right)-\frac{A2}{A1}\;\left(Control\;cells\right)\times 100$$

*A*1 represents the gap width at 0 time; *A*2 represents the gap width after 48 h or 72 h.

### 4.7. Human Angiogenesis Antibody Array

The effect of fucoxanthin on 43 cytokine proteins produced by TNF-activated TNBC cells was investigated using RayBiotech human angiogenesis antibody arrays. The experiments were carried out in triplicate and as the manufacturer’s protocol instructed. The antibody-coated array membranes were incubated with 2 mL of blocking buffer for 30 min at room temperature. The blocking buffer was aspirated from each well. Next, 1 mL of the sample, exposed to different treatments, was added to each well and incubated for 24 h at 4 °C with slow, gentle rocking. Treatments consisted of TNF-α (50 ng/mL) and the combination of fucoxanthin (6.25 µM) + TNF-α (50 ng/mL). The following day, the supernatants were aspirated; membranes were washed and incubated with 1 mL of biotinylated antibody cocktail for 2 h with slow, gentle rocking. The biotinylated antibodies were aspirated, and membranes were washed and incubated with HRP-conjugated streptavidin for 2 h. Lastly, 500 µL of chemiluminescence detection buffer was added to each membrane and incubated for 2 min in the dark. The images of the membranes were exposed and captured by the Max Multi-imager (Bio-Rad Laboratories, Hercules, CA, USA), and the spot density was determined with Quantity One Software (Bio-Rad Laboratories, Hercules, CA). Analysis by Excel-based data was performed using Human Angiogenesis Antibody Kit software C100 (CODE: S02-AAH-ANG-1000-4) from RayBiotech.

### 4.8. Real-Time Polymerase Chain Reaction (RT-PCR)

#### 4.8.1. RNA Extraction

After 24 h of exposure to various treatments, the cells were harvested, and cell pellets were collected. The treatments comprised the control (cells + DMSO), fucoxanthin (6.25 μM), TNF-stimulated (50 ng/mL), and co-treated with fucoxanthin (6.25 μM) + TNF (50 ng/mL). Then, 1 mL of TRIzol reagent was added to each homogenized pellet (Thermo Fischer Scientific, Wilmington, DE, USA). Next, 200 µL of chloroform (Sigma-Aldrich Co., St. Louis, MO, USA) was added to each lysed sample; the samples sat in a rack for 3–5 min at room temperature, were vortexed for 15–30 s, and centrifuged for 12 min at 11,000 rpm at 2–8 °C. A set of clean tubes with 500 µL of isopropyl alcohol (Sigma-Aldrich Co., St. Louis, MO, USA) was used to collect the clear aqueous phase for each sample. For RNA precipitation, the tubes were inverted and incubated at room temperature for 10 min, and then centrifuged for 12 min at 11,000 rpm at 2–8 °C. The supernatant was removed, and the RNA pellets were washed with 75% ethanol (inverted carefully) and centrifuged for 5 min at 9000 rpm at 2–8 °C. After centrifuging, ethanol was carefully aspirated without disturbing the RNA pellet, and all tubes were placed for 15 min at room temperature to evaporate ethanol droplets. The RNA pellet was dissolved in 35 µL of RNase-free water and incubated on ice for 30 min. Finally, the Nanodrop (Thermo Fisher Scientific, Wilmington, DE, USA) was used to determine RNA quantity and purity.

#### 4.8.2. RT-PCR and cDNA Conversion

Reverse transcription was performed using the thermocycler program, and transcription steps included 46 °C for 20 min and 95 °C for 1 min. Next, a combination of 1 µL of the sample (200 ng cDNA/reaction), 8 µL of water, 1 µL of primer, and 10 µL of SybrGreen were combined and added to each well. RT-PCR was performed at 95 °C for 2 min, denaturation at 95 °C for 10 s, followed by 39 cycles of 60 °C for 30 s (annealing/extension), and 65–95 °C for 5 s/step (melting curve) using the Bio-Rad CFX96 Real-Time System (Hercules, CA, USA). The following primers were used: VEGF-C (qHsaCID0015147), GAPDH (qHsaCED0038674), and VEGF-A (qHsaCED0006937).

### 4.9. Data Analysis

Statistical analysis was performed using GraphPad Prism (version 6.07). All data were expressed as mean ± SEM from at least three independent experiments, and the significance of the difference between the two groups was assessed using a Student’s *t*-test; meanwhile, one-way ANOVA was used for more than two groups, followed by Dunnett’s multiple comparison test. * *p* < 0.05, ** *p* < 0.01, *** *p* < 0.001, and **** *p* < 0.0001, ns = *p* > 0.05.

## 5. Conclusions

Fucoxanthin showed higher cytotoxicity in MDA-MB-468 cells compared to MDA-MB-231 cells. The compound had similar antiproliferative effects on both MDA-MB-468 and MDA-MB-231 cells. It induced cell cycle growth arrest and had an inhibitory effect on cellular migration in both cell lines. Fucoxanthin only induced apoptosis in MDA-MB-231 and had no apoptotic effect on MDA-MB-468 cells. However, there was a greater significant inhibitory effect on cellular migration in MDA-MB-231 cells after 48 h compared to MDA-MB-468 cells after 72 h, showing that the compound is more effective in MDA-MB-231 compared to MDA-MB-468 cells.

**Figure 11:**
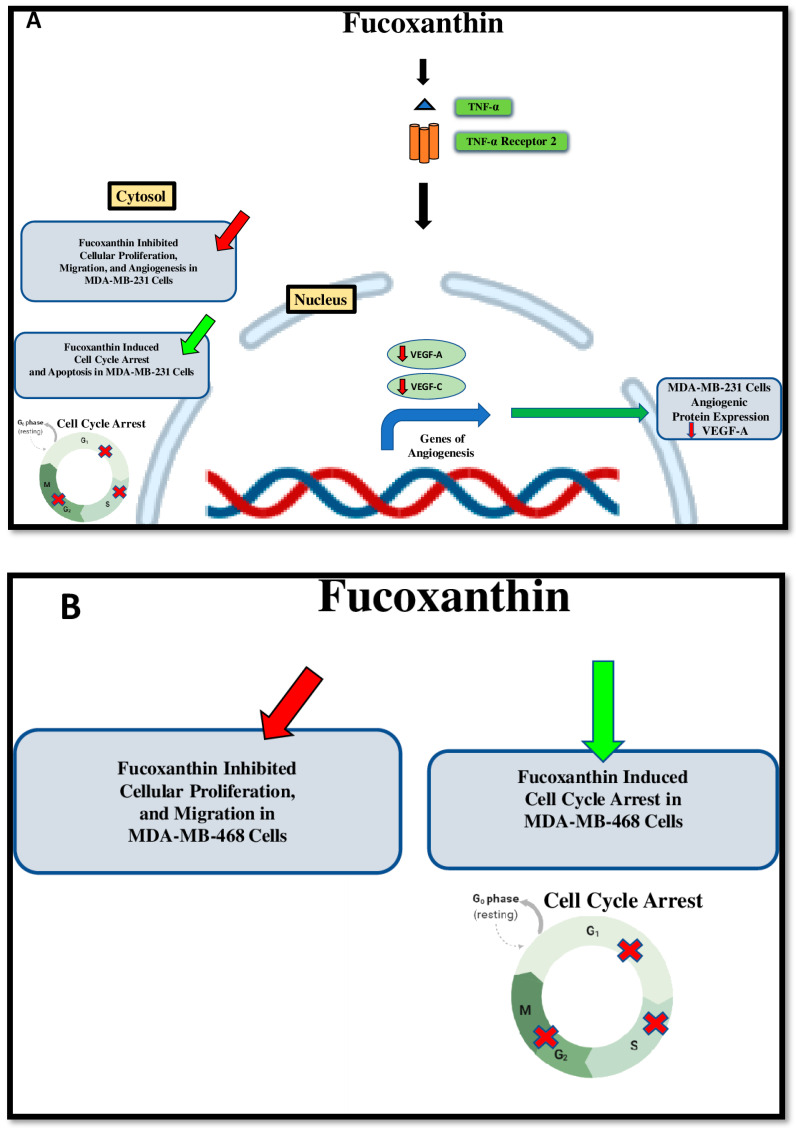
Proposed fucoxanthin inductive effects on several cellular processes and modulatory effects through TNF-α stimulation in MDA-MB-231 (**A**) and MDA-MB-468 (**B**) cells. The green arrow shows that activation is induced by fucoxanthin. Red arrows show that fucoxanthin induces inhibition, and black arrows indicate the fucoxanthin modulatory effect.

Moreover, the compound inhibited the expression of VEGF-A in the MDA-MB-231 cell line, but not in the MDA-MD-468 cell line. The results show a diverse response of MDA-MB-231 compared to MDA-MB-468 cells after fucoxanthin treatment (Figure 11A,B). In conclusion, this study shows that fucoxanthin was more effective in MDA-MB-231 TNBC, where it can target VEGF-A and VEGF-C, inhibit cell proliferation, cell migration, and induce cell cycle arrest and apoptosis, which are cellular processes involved in breast cancer development and progression. The obtained results urge further investigation on MDA-MB-468 cells. Also, in vivo studies should be considered in the future.

## Data Availability

All data from this study are included in this published article.
